# Heterogeneous catalytic activation of peroxydisulfate toward degradation of pharmaceuticals diclofenac and ibuprofen using scrap printed circuit board†

**DOI:** 10.1039/d2ra07263g

**Published:** 2022-12-20

**Authors:** Kosar Hikmat Hama Aziz

**Affiliations:** Department of Chemistry, College of Science, University of Sulaimani Qlyasan Street Sulaimani City 46001 Kurdistan Region Iraq kosar.hamaaziz@univsul.edu.iq; Department of Medical Laboratory of Science, College of Health Sciences, University of Human Development Sulaimani Iraq

## Abstract

Pharmaceutical residues have been identified as a priority contaminant due to their toxicity to organisms and the ecosystem as representative refractory organic compounds in water. Therefore, using efficient treatment methods to remove them from wastewater has become a crucial topic of research. Advanced oxidation processes (AOPs) based on the sulfate radical have gained increased attention in recent years due to their superior performance and adaptability in the decomposition of refractory organic contaminants. In this work, scrap printed circuit boards (PCBs) were used to prepare a low-cost and efficient heterogeneous peroxydisulfate (PDS) catalytic activator *via* thermal treatment with an air combustion non-carbonized catalyst (NCC) and pyrolysis with a nitrogen carbonized catalyst (CC) for the removal of diclofenac (DCF) and ibuprofen (IBF) from water at circumneutral pH. The synthesized catalysts were characterized by several analytical techniques. The effects of various experimental parameters on the removal efficiency were examined. Under optimum conditions, the degradation efficiency reached 76% and 71% with NCC and 63% and 57.5% with CC within 60 min for DCF and IBP, respectively. The mineralization efficiency as measured by TOC removal reached up to 65% after 60 min treatment. The degradation kinetics for both catalysts followed the pseudo-first-order model. Results from quenching tests showed that the reactive oxidizing species (ROS), including ^1^O_2_ > SO_4_˙^−^ > ˙OH, were generated mainly in the NCC/PDS and CC/PDS systems. Overall, the prepared catalysts were found to be effective and reusable for PDS activation for the removal of pharmaceutical pollutants from water. This study provided a promising, robust and efficient heterogeneous catalytic PDS activation based on the strategy of “waste-treats-waste” for the removal of pharmaceutical pollutants from water.

## Abstract 2

Scrap printed circuit boards were used to prepare a low-cost and efficient heterogeneous peroxydisulfate catalytic activator for the removal of diclofenac and ibuprofen from water at circumneutral pH.

## Graphical abstract

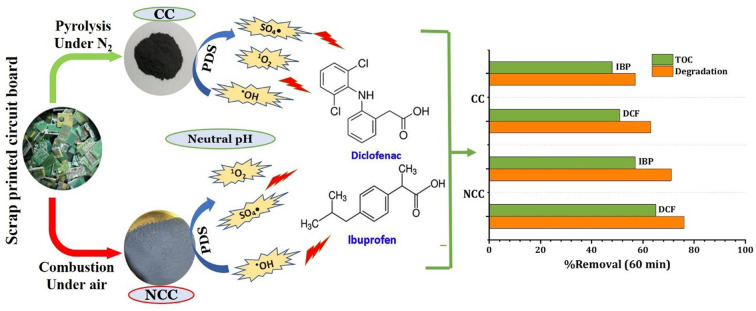

## 1. Introduction

The occurrence of organic pollutants in wastewater has increased tremendously in recent years and has become a critical concern because of their toxicity, semi-volatile nature, refractory behavior, high bioaccumulation, and non-biodegradability under normal environmental conditions.^1,2^ Pharmaceutical and personal care products are inextricably discharged into the aquatic environment due to the rapid development of the pharmaceutical industry and rising drug usage. Pharmaceuticals in wastewater, particularly hospital wastewater, are a major environmental concern because they are not removed by conventional wastewater treatment processes and are discharged into aquatic systems.^3^ Diclofenac (DCF) and ibuprofen (IBP) are anti-inflammatory drugs used to treat toothaches, rheumatoid arthritis, muscle aches, and migraines, as well as analgesics and antipyretics.^4^ Pharmaceutical residues discharged into the aquatic environment from various sources such as the textile industry, hospitals, institutes, and industrial plants cannot be controlled as a result of population growth, posing a severe threat to ecosystems and human health worldwide.^5,6^ The concentration of pharmaceutical residues in natural water is usually between μg L^−1^ and ng L^−1^ but it is recognized as a potential risk for aquatic ecosystems because of the long-term negative effects on aquatic organisms and humans intestines.^7^ Most pharmaceuticals and personal care products are toxic, and bio-refractory compounds that can hardly be treated by conventional wastewater treatment methods. According to the reported research, DCF and IBP constituted a serious threat to both human health and the aquatic ecosystem, particularly fish.^8^ The removal of pharmaceutical pollutants from water is important for the sustainability of natural life. Therefore, before releasing these medications into the aquatic environment, it is vital to discover efficient methods to remove or degrade them from water.^9,10^ In this regard, water purification has now become the main critical issue worldwide for which strict legislation has been formulated by World Health Organization for organic compounds for Drinking-Water Quality.^11^ Many ongoing studies in water purification remain a severe challenge to governments, scientists, and industries, as the lack of cost-effective water purification technology, has aggravated the crisis of clean and safe water for the fast-expanding population. Over the years, various methodologies to remediate water threats from toxic organic pollutants have been developed, such as photocatalytic degradation, chemical oxidation, micellar enhanced ultra-filtration, advanced oxidation, aerobic degradation, adsorption, filtration, ozonation, coagulation, flocculation, distillation, extraction, precipitation.^12^ Numerous techniques are currently being studied to eliminate pharmaceuticals from wastewater. Advanced oxidation processes (AOPs) are considered effective methods for remediating refractory organic pollutants from water, owing to their high reactivity and oxidation capability of a wide range of contaminants.^13,14^ Bio-resistant and recalcitrant organic pollutants can be completely decomposed by advanced oxidation processes. These methods are mainly based on the production of reactive oxygen species such as non-selective and highly reactive hydroxyl radicals and sulfate radicals in sufficient quantity to oxidize and mineralize refractory organic contaminants into carbon dioxide, water, and inorganic acids at ambient temperatures.^15–17^ Sulfate radicals-based AOPs have gained increasing attention of many researchers as a simple and effective way for degradation and mineralization of recalcitrant organic pollutants in water.^18^ In such methods, peroxymonosulfate (PMS) or peroxydisulfate (PDS) are commonly activated to produce sulphate radicals. Degradation of pharmaceutical DCF and IBP from water was previously studied using photocatalysis in falling film reactor,^19^ non-thermal plasma generated by dielectric barrier discharge under different gas atmosphere and ozonation alone or in combination with photocatalysis^20^ and various AOPs.^4,21,22^ Catalysts that are low-cost, efficient, and environmentally safe are preferred for heterogeneous catalytic persulfate activation. Many studies have focused on the utilization of waste-to-resource strategies to manufacture an efficient catalyst for environmental remediation using agricultural and industrial wastes as a raw material.^23^ Electronic waste disposal and reuse have become a major global concern. Thus, the utilization electronic waste materials for synthesis of efficient and low-cost catalyst to reduce environmental impact have reported as global interest in many researches.^24–26^ Hydroxyl-based AOPs, degrade organic pollutants in a non-selective multi-step route that typically requires an acidic environment. However, the hydroxyl radical is non-selective, matrix components in water like common anions and natural organic materials can easily scavenge it. Interest in the oxidation of organic contaminants by other reactive species, such as the sulfate radical, which are less affected by anions and natural organic materials present in water, has recently increased. The common chemicals that can be activated to generate sulfate radicals (SO_4_˙) are persulfate, including PMS and PDS in sulfate-based AOPs.^27^ Various activators such as heat, transition metal catalysts, carbonaceous materials, electrochemical activation, alkaline conditions, powerful oxidants, and ultraviolet (UV) radiation can all be used to produce sulfate radicals from PDS or PMS activation *via* cleavage of O–O bond (reaction R(1) and R(2)).^14,28^ Sulfate radical has a high redox potential and selectivity and is a strong single electron oxidant. It is more easily diffused in the complex environmental matrix than hydroxyl radical due to its longer half-life.^29,30^ In the presence of naturally occurring organic materials, such as humic acid, which is primarily found in natural water, the efficiency loss in sulfate radical-based AOPs is significantly lower than that in OH-radical-based AOPs.^31^

R1 S_2_O^-2^_8_ + activator → SO^−^_4_˙ + (SO^−^_4_˙ or SO^−^_4_)

R2 HSO_5_^−^ + activator → SO^−^_4_˙ + (HO˙ or HO^−^)

Among the treatment processes studied, sulfate radical-based AOPs proved to be promising methods for refractory organic pollutants degradation because sulfate radical is more oxidizing than hydroxyl radical, and it may act in a wider pH range and lasts longer in aqueous solutions.^27,32^ As a PDS activator, transition metal and carbon-based catalysts are intensively studied. One electron is transferred from the metal-based catalyst to PDS, resulting in the formation of the sulfate radical.^33,34^ Although transition metal catalysts based on Fe, Co, Zn, Cu, and Ag have been reported as efficient PDS activators for the removal of organic pollutants, copper-based catalysts are currently dominating research. This is mainly due to the wider working pH range, cost-effective, eco-friendly (less toxic and bioavailable), and more reactive compared to other transition metal-based catalysts.^13,35^ In the degradation of aqueous naproxen, the activation of PMS using standard-sized copper sheet and copper foam in the presence and absence of a graphene layer was examined.^36^ The degradation of DCF by AOP based on iron(ii) in combination with chlorine and peroxymonosulfate was compared at optimized initial solution pH values of 3 and 4.^37^ The application of zero-valent copper and copper oxide catalysts for persulfate activation in the presence of UV for sulfamerazine degradation in an aqueous solution was investigated in.^38^ The photocatalytic and persulfate activities of graphene oxide composited with copper antimony sulfide nanoparticles for the degradation of tetracycline were studied.^39^ Through carbothermal reduction technique, porous silicate supported micro-nano zero-valent iron was synthesized from copper slag and employed as a persulfate activator for eliminating organic pollutants.^40^ CuCo/carbon generated from metal–organic frameworks was synthesized and evaluated as an efficient magnetic heterogeneous catalyst for persulfate activation and ciprofloxacin degradation.^41^ The activation of PDS for organic pollutants degradation by CuO nanoparticles based on layered MgO was also investigated.^42^ Copper-based catalysts as persulfate activated are discussed and summarized in.^32^

These approaches are efficient and effective in the treatment of wastewater. However, most of these catalysts require a complex, expensive, and unsafe manufacturing procedure. In this work, low-cost and efficient persulfate activator catalysts have been synthesized by a facile and green method using scrap PCBs based on the “waste-to-resource” strategy. The catalytic activity of the synthesized catalysts (NCC and CC) in heterogeneous PDS activation was studied by using DCF and IBP as model contaminants. The effect of various experimental conditions on the DCF and IBP degradation efficiency was investigated and optimized. The mineralization efficiency tested by TOC removal was also examined. The kinetic investigation was carried out in optimal conditions, and the reaction order was determined based on the best fit. The application of the prepared catalysts as PDS activators has shown the expected results, which provides a new approach for the removal of pharmaceutical residues from wastewater at circumneutral pH.

## 2. Material and methods

### 2.1. Materials

The chemicals and solvents used in this research were high purity and used as received. The stock solution of DCF and IBP were prepared by dissolving their respective sodium salts obtained from Alfa Aesar (purity > 98.5%, Germany) and Fluka (>98% GC), respectively. Sodium persulfate (>99% purity) was purchased from Sigma Aldrich. Deionized water was used throughout the work to prepare all solutions. Electronic waste of scrap PCBs was collected from local computer service shops. The sodium salt of the drugs was used to prepare the stock solution of DCF and IBP (250 mg L^−1^) in deionized water.

### 2.2. Characterization of synthesized catalysts

EDXRF-S2 PUMA, SEM-EDS (Bruker, Germany), X-ray photoelectron spectrometry (XPS, Thermo Fisher, USA), nitrogen adsorption–desorption isotherms (Micromeritics, USA), Fourier transform infrared spectroscopy (FT-IR, Shimadzu, Japan) and Raman spectrometer (Lab Ram HR, Horiba) with a laser intensity of 532 nm wavelength and an acquisition time of (16 ms to 10 min) were used to characterize the prepared CC and NCC Fenton like catalysts. Thermal stability was determined by using a thermogravimetric (TG) analyzer (STA 503) (BAHR Germany) from (45–820 °C) at a heating speed of (0.01–100 K min^−1^). The zeta potential of the catalyst was evaluated using a Zeta potential analyzer (SZ-100, Horiba). Magnetic properties of the synthesized catalysts were analyzed using a vibrating sample magnetometer (MDKB).

### 2.3. Experimental setup and procedure

The prepared catalysts NCC and CC were synthesized *via* two different procedures.^26^ PCBs were collected from a computer repair shop. After removing the electronic components such as capacitors, electrical resistor, CPU, and connecter the remaining parts of the PCB were washed and dried at room temperature. Next, the samples were cut into (2–4 cm) pieces. For CC catalyst: the obtained PCB was crushed by a high-speed crusher model-100 and the powder was put into a ceramic crucible following pyrolysis in a tubular resistance furnace under nitrogen at a flow rate of 2 L min^−1^ at 500 °C for 2 hours. Finally, the prepared catalyst cooled to room temperature under nitrogen for 10 min to avoid oxidation, the carbonized PCB was ground in an agate mortar and sieved into 70 mesh for application in the degradation processes. For NCC catalyst: for NCC: the CPB was burned in the presence of air and then crushed. It was then placed in a crucible and ignited for 6 hours at 650 °C in a Muffle furnace. It was then brought to room temperature, mortar-crushed, and sieved into 70 meshes. The yield of CC and NCC were calculated as 80 and 60%, respectively.

All degradation experiments were carried out in batch mode. The applicability of synthesized catalysts was investigated *via* batch experiments in a 250 mL glass vessel containing 100 mL of 20 mg L^−1^ DCF or IBP at optimum reaction conditions. Next, the reactions were started by adding a certain amount of PDS to a mixture solution of drugs with CC or NCC. The solution was shaken by KIKA-WERKE shaker (model: KS501 digital). After that, at a specific time, about 2 mL of the sample was taken and filtrated by cellulose acetate membrane filter by CHMLAB Group (0.45 μm, Cat No: SCA045025K-S) to remove the solid catalyst. The solution was then transferred into an HPLC vial for analysis. The observed DCF and IBP degradation process was well described by the pseudo-first-order kinetic model. The batch experiments were performed in duplicate at room temperature (about 21 °C) and the average results were taken.

### 2.4. Analysis

The concentrations of DCF and IBP were measured using high-performance-liquid chromatography (HPLC; Shimadzu, LC-2030C plus) equipped with an ultraviolet detector and an analytical column: NUCLEODUR 100-5 C18 (250 mm × 4.6 mm, 5 μm pore size, Agilent). The injection volume was 10 μL and the flow rate was 1.0 mL min^−1^. The eluent conditions were 40% 0.1 M sodium acetate and 60% methanol solution for DCF and 40% 0.1 M chloroacetic acid and 60% acetonitrile for IBP keeping for 30 min at 254 and 264 nm, respectively. Leached metal ions in the aqueous solution after treatment reaction was measured using inductively coupled plasm atomic emission spectroscopy (ICPE-9820, Shimadzu). Total organic carbon (TOC) removal was used to calculate the mineralization efficiency using a TOC-Analyzer (TOC-VCPH, Shimadzu).

## 3. Results and discussion

### 3.1. Characterization of fabricated catalysts

**Fig. 1:**
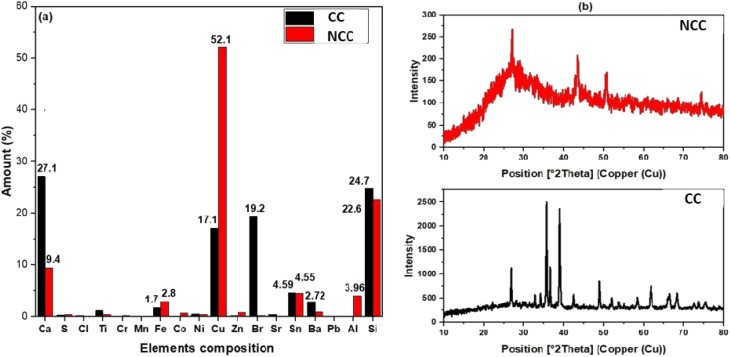
(a) The results of EDXRF and (b) XRD spectrum.

The elemental composition of the prepared catalysts was examined using X-ray fluorescence; as can be seen in Fig. 1a, copper is the predominant transition metal in both catalysts, with a minor amount of iron. The main active element for the activation of PDS in could be copper in the forms of Cu, Cu_2_O, and CuO (Fig. 1a). The crystal structure of the prepared catalysts (NCC and CC) was characterized by X-ray diffraction patterns (XRD). XRD had diffraction peaks at 30° to 70° that were well matched to the crystal planes of monoclinic CuO (JCPDS 45-0937).^43,44^ The main constituents of the CC and NCC catalysts are oxides of base metals including CuO, SnO_2_, CaO, FeO, and SiO_2_. A peak around 25° typically represents a graphite-like carbon structure, while graphite-like carbon, with a higher intensity in CC, is represented by the peak around 43.5° (Fig. 1b).^45^ CC has a stronger peak at 43.5° than NCC, demonstrating a higher degree of graphitization in CC. The monoclinic and cubic crystal system diffraction peaks at 36°, 42.5°, 49°, 53.5°, 61.5°, and 73° confirm the existence of Cu, Cu_2_O, and CuO in both catalysts.^46^

**Fig. 2:**
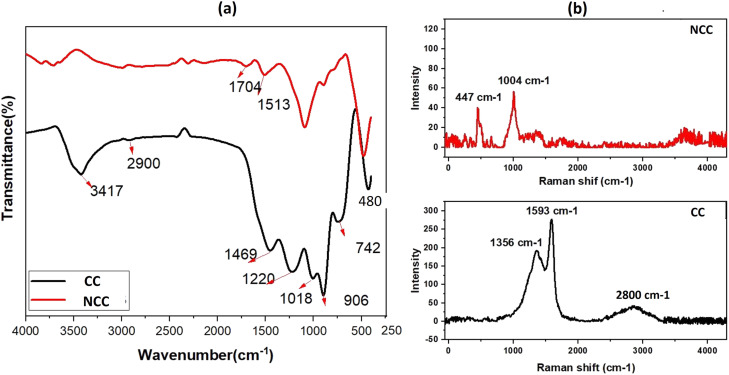
(a) FTIR spectra and (b) Raman spectra of the prepared catalysts.

The FTIR spectra of prepared catalysts are illustrated in Fig. 2a. The stretching vibrations of the –OH can be attributed to the large peak at about 3417 cm^−1^. This broad peak (black) reveals the presence of OH bonds in the CC catalyst. The presence of Cu–O stretching is confirmed by the peaks at 480 cm^−1^, indicating the presence of CuO in both catalysts.^47^ The peak at 1220 cm^−1^ is ascribed to C–O–C and the vibrational bands which correspond to –CH and –CH_2_ groups, are observed at about 2900 cm^−1^ in CC.^48^ The peak at 1469 cm^−1^ is ascribed to C–H bending. The Raman spectrum for the CC catalyst reveals three dominant peaks at 1356, 1593, and 2800 cm^−1^, which are contributed by the D, G, and 2D bands of carbon and are typical peaks for graphitic carbon structure.^49^ The peak appeared at 1004 cm^−1^ assign to B_2g_ peak of CuO in NCC catalyst. The intensity ratio between the D-band and G-band is used to determine the degree of carbon defect. The calculated ratio for CC catalyst was 0.7. The NCC catalyst has a minor peak at 447 cm^−1^ and 1004 cm^−1^, with intensities of 40.2 and 56.49, indicating a low concentration of carbon and carbon compounds.

**Fig. 3:**
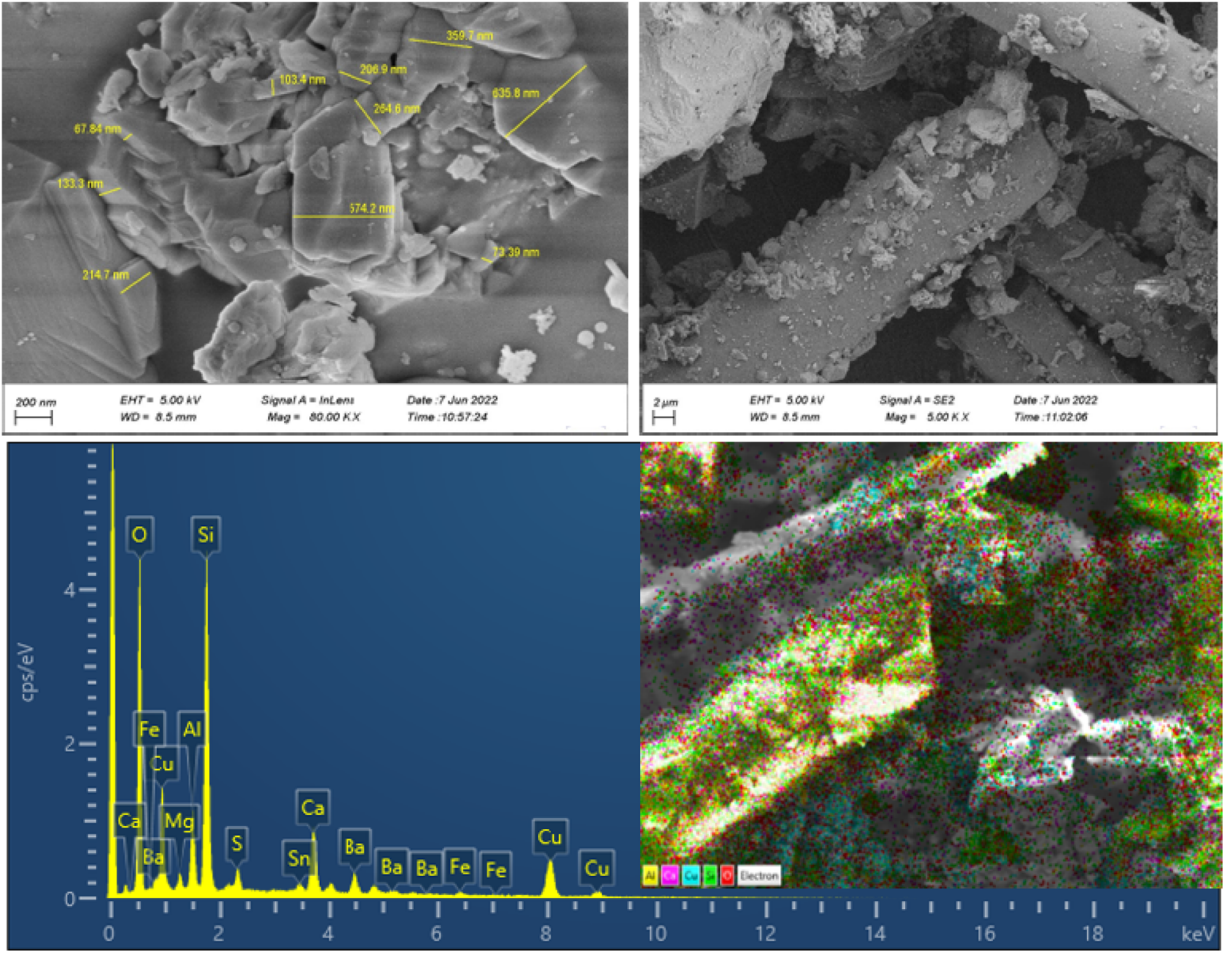
SEM-EDS results of NCC.

**Fig. 4:**
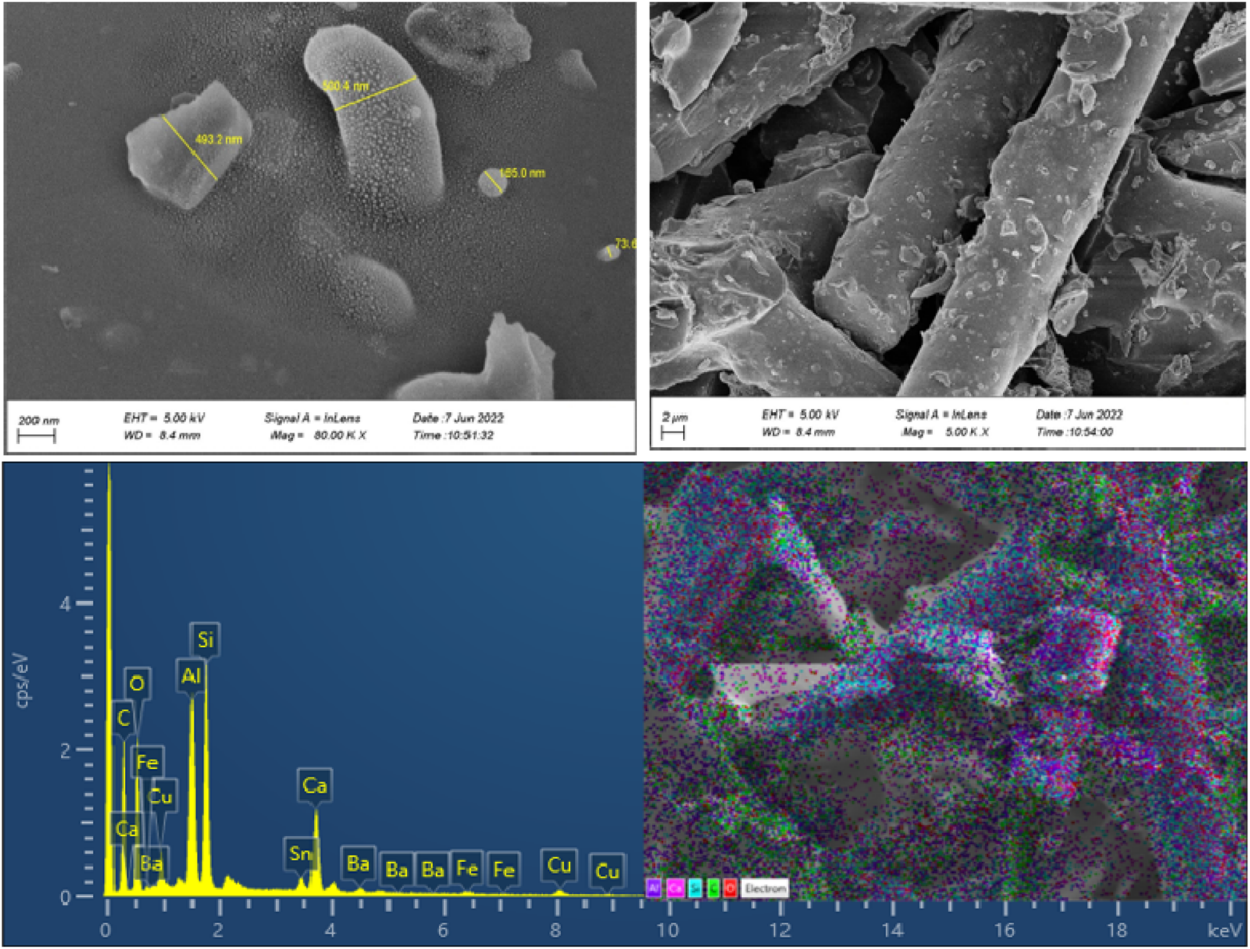
SEM-EDS results of CC.

The morphologies, particle size distribution, and surface elemental mapping of the prepared catalysts were characterized by scanning electron microscopy in combination with energy-dispersive X-ray spectroscopy (SEM-EDS). Fig. 3 and 4 show the results of SEM and EDS images of the prepared catalysts with different magnifications. The SEM images in Fig. 3 and 4 showed that the granular particles and rod-shaped structures were composed of a variety of micro-sized (up to 10 μm) to submicron-sized (70 to 600 nm) metal oxides and metals. The mapping images of prepared catalysts also confirm the presence of the main elements, including C, Al, Si, Fe, Cu, and O. further information about the elemental mapping and composition of both catalysts can be found in ESI.†

**Fig. 5:**
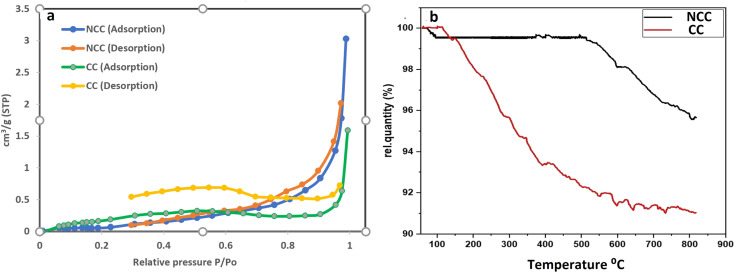
(a) BET (N_2_ adsorption–desorption) isotherm curves of the synthesized catalysts and (b) Thermogravimetric analysis (TGA).

The surface area and pore characteristics of the prepared catalysts were next studied using the BET and BJH techniques, respectively, using N_2_ adsorption–desorption isotherms. The isotherm curves of the synthesized catalysts (NCC and CC) are shown in Fig. 5a. Based on these curves, it was shown that the isotherm curve of the synthesized catalysts was assigned to Type II according to the IUPAC category. The results demonstrate microporous adsorption–desorption behavior with a hysteresis loop. Larger surface areas generally expose more active sites, increasing surface adsorption capacity and catalytic effectiveness. On the other hand, more open pores and larger porosity would make it easier for reactants and products to enter and exit the active regions. The data on the catalytic surface area, pore volume, and pore size of the synthesized catalysts can be seen in Table 1. The average BJH pore size distribution of both catalysts is less than 2 nm, indicating microporous adsorption–desorption behavior. NCC catalyst has a lower surface area (0.21098 m^2^ g^−1^) than CC (1.2953 m^2^ g^−1^), which may be due the presence of high carbon content in CC.

**Table 1:** BET surface area, total pore volume, and average pore diameter of the fabricated catalysts

Catalysts	*S*_BET_ (m^2^ g^−1^)	Pore volume (cm^3^ g^−1^)	Average pore diameter (nm)
CC	1.295	0.002197	1.21
NCC	0.211	0.004687	1.85

TGA is a helpful tool for evaluating the thermal stability of catalysts. The mass change of the catalysts is determined after increasing the temperature. Fig. 5b displayed the TGA values for the synthesized catalysts. According to the results there were two stages to weight loss. The first step involved mass loss due to the removal of adsorbed water and took place between room temperature and 160 °C. The second stage at 160–800 °C may be associated with organic material bond ruptures in PCBs. Results from the TGA revealed that NCC is more stable than CC over a wide range of temperatures and that CC contains more carbon compounds than NCC.

**Fig. 6:**
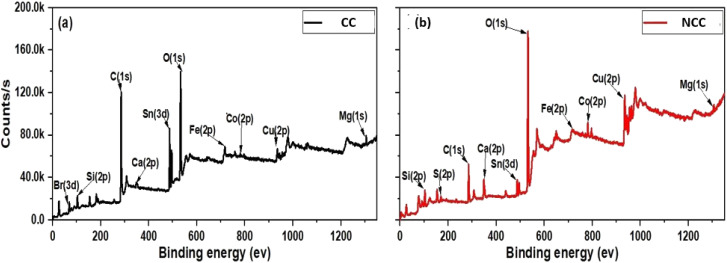
X-ray photoelectron spectroscopy (XPS) (a) CC and (b) NCC.

The discrepancies between the chemical compositions and elemental states of the CC and NCC catalysts were clarified using XPS. As shown in Fig. 6 XPS spectrum of Cu (2p) has appeared at 933–952 eV (Cu(0) or Cu(i)) and Cu(ii), 943 eV,^44^ The two peaks of about 933 and about 952 eV arising from Cu(i) or Cu(0) and the peaks at 943 appear from Cu(ii). The results demonstrated that Cu(0), Cu(ii), and Cu(i) existed in prepared catalysts.

**Fig. 7:**
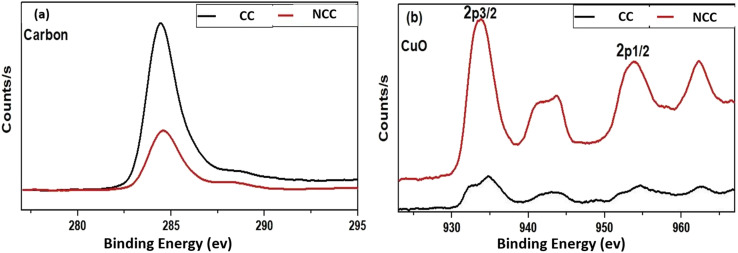
X-ray photoelectron spectroscopy (XPS) (a) Carbon and (b) CuO.

The XPS spectra of the Cu(2p) spectrum are shown in Fig. 7, which shows that NCC has more copper than CC catalyst. However, as shown in Fig. 7a, the carbon signal on the catalytic surface of CC is much higher than that of NCC, indicating that CC has a higher carbon content than NCC. The detailed XPS results can be seen in the ESI.†

**Fig. 8:**
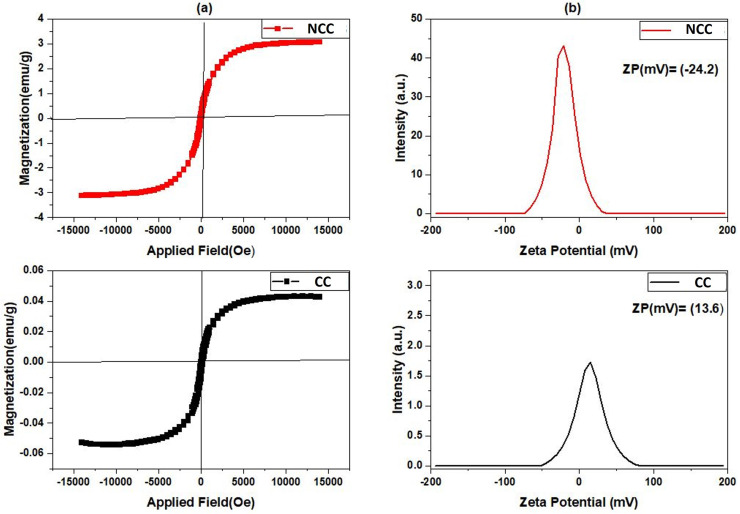
(a) VSM, (b) Zeta potential.

The most popular tool for analyzing the magnetic characteristics of Cu-based catalysts is the Value Stream Mapping (VSM) device. The magnetic hysteresis loops of produced catalysts were measured at room temperature with a VSM in the field range of −15 K(Oe) to −15 K(Oe), and the results are shown in Fig. 8a. The maximum magnetization saturation value of NCC catalyst was 3.077 emu per g, which was higher than that of CC catalyst (0.042 emu per g). The presence of more nonmagnetic carbon compounds, and pyrolysis conditions during the CC preparation process may have contributed to the decrease in magnetization saturation. According to the results obtained from VSM studies an external magnetic field may easily separate the NCC catalyst from the treated solution, making them useful in separation science and technology. For examining the magnetic properties of synthesized catalysts, zeta potential as a common technique was used. According to the results presented in Fig. 8b, the zeta potential (ZP) of NCC catalyst is negative, whereas CC catalyst has a positive zeta potential. It is well known that the pH of Cu-based catalysts at PZC values correlates with their acidic/basic features and can be used to estimate the surface charge of the catalysts in each pH solution.^50^ The thermal process condition involved in the catalyst production is responsible for the difference in ZP, which is −24.6 mV in the case of combustion under air for NCC, and 13.6 mV in the case of pyrolysis under N2 for CC. The points of zero charge (PZC) of examined catalysts in aqueous solution can be found using zeta potential analysis. The results from Fig. 8 revealed that the PZCs for NCC and CC catalysts are found at 4.7 and 7.2, respectively.

### 3.2. Degradation and mineralization of DCF and IBP

**Fig. 9:**
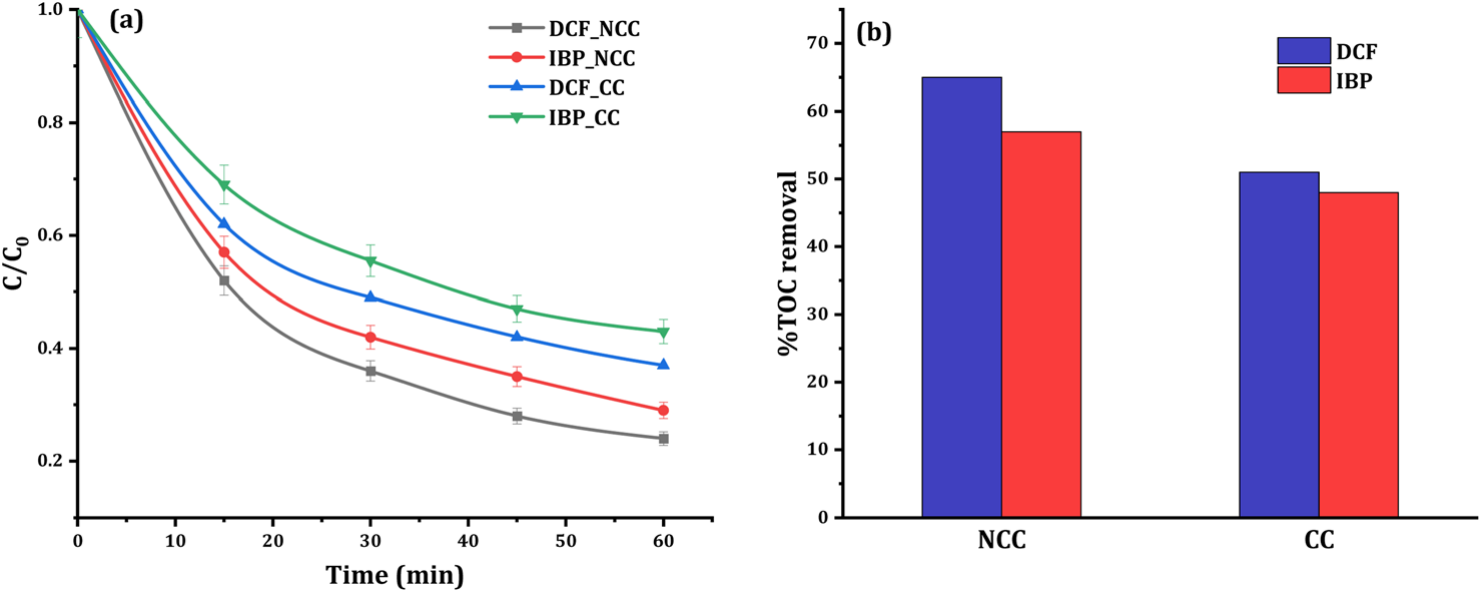
Removal of DCF and IBP (a) relative degradation profile, (b) TOC removal within 1 hour: (experimental conditions: 0.1 L [DCF]_o_ or [IBP]_o_ = 20 mg L^−1^, [PDS]_o_ = 6 mM, catalyst doses = 0.75 g of CC or NCC).

Control studies in the dark without PDS were carried out to see if adsorption processes played a role in the removal efficiency. After 60 minutes of circulation, insignificant changes in the concentrations of 20 mg L^−1^ DCF or IBP were observed, indicating that adsorption mechanisms are not involved in the elimination process. In contrast, the reductive degradation reaction commences quickly after the addition of PDS to the solution mixture of pharmaceuticals with catalysts, which are identified from the HPLC chromatogram data. The HPLC chromatograms before and after the degradation of DCF and IBP were shown in the ESI (Fig. S3 and S4†). The removal efficiency of DCF and IBP using CC and NCC catalysts was compared in the presence of 20 mg L^−1^ pollutant, 6 mM PDS, and 0.75 g catalyst at a neutral initial pH. It was observed that there was no significant pH change in the solution during the treatment processes. The degradation profiles and degree of mineralization of both pharmaceuticals are shown in Fig. 9a. The obtained results confirmed the catalytic activity of the CC and NCC catalysts in the activation of PDS *via* generation sulfate and hydroxyl radicals towards the degradation of DCF and IBP. The mineralization efficiency can be used to measure the effectiveness of any wastewater treatment based AOP. Poor mineralization means that the applied technology is still leaving some refractory organic byproducts in the system, which may be more toxic and destructive than initial contaminants. Therefore, high total organic carbon (TOC) removal is an important key to the successful wastewater treatment system by AOP. After the 1 hour of treatment, the TOC concentration was also examined. Under optimum conditions, the removal of TOC at 20 mg L^−1^ DCF and IBP was determined to be 65% and 57% using NCC and 51% and 48% using CC, respectively. The results of mineralization efficiency measured by TOC removal for both pharmaceuticals are shown in Fig. 9b. N-doped graphene aerogel has been used as peroxymonosulfate activator for oxidative degradation of ibuprofen. The catalytic activities of the synthesized catalyst was reached up to 90% IBP (20 mg L^−1^) degradation after 180 min.^51^ The activation of peroxymonosulfate by BiFeO_3_ microspheres has also been used to remove DCF in the aqueous phase. The activation of peroxymonosulfate by BiFeO_3_ degraded 47% of DCF within 60 min.^52^ The results obtained in this work suggested that the synthesized catalysts could have a promising potential in activating PDS to degrade pharmaceutical pollutants in water.

### 3.3. Optimization of experimental parameters and degradation kinetics

**Fig. 10:**
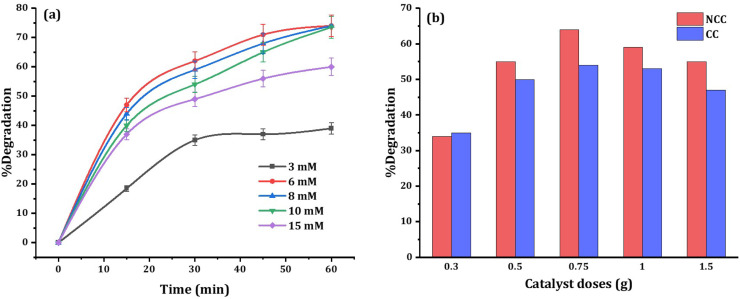
Effects of (a) PDS dosage, and (b) catalyst dosage on the 20 mg L^−1^ DCF degradation at circumneutral pH.

To enhance the efficacy of the prepared catalysts, experimental parameters including PDS dosage and catalyst dosage were examined. The effect of PDS dosage (3, 6, 8, 10, and 15 mM) on DCF degradation was investigated at neutral initial pH and the results are presented in Fig. 10a. High concentration of PDS or high catalyst dose has a generated reactive radical scavenging effect.^53^ The production of more reactive species in the solution is facilitated by an increase in PDS concentration, but when the concentration surpasses the optimal level, scavenging effects reduce the efficiency (Fig. 10a). Therefore, to enhance the degradation efficiency, and considering the economic cost, 6 mM PDS was selected as optimum initial concentration in all experiments. When the PDS dosage rises from 6 mM to 15 mM, the degradation of DCF does not increase significantly, which may be due to excessive PDS occupying the active sites of catalysts. The amount of catalyst used is also important for both the production of reactive species and the degradation of DCF. As shown in Fig. 9 the optimum reaction condition was selected to be 6 mM PDS, and 0.75 g NCC or CC catalysts at neutral pH. However, with further increase in PDS concentration, or catalyst doses the pollutant removal efficiency decreased due to the competition and adverse reaction between PDS or excess catalysts with produced ˙OH and SO_4_˙ in the solution.^54^

**Fig. 11:**
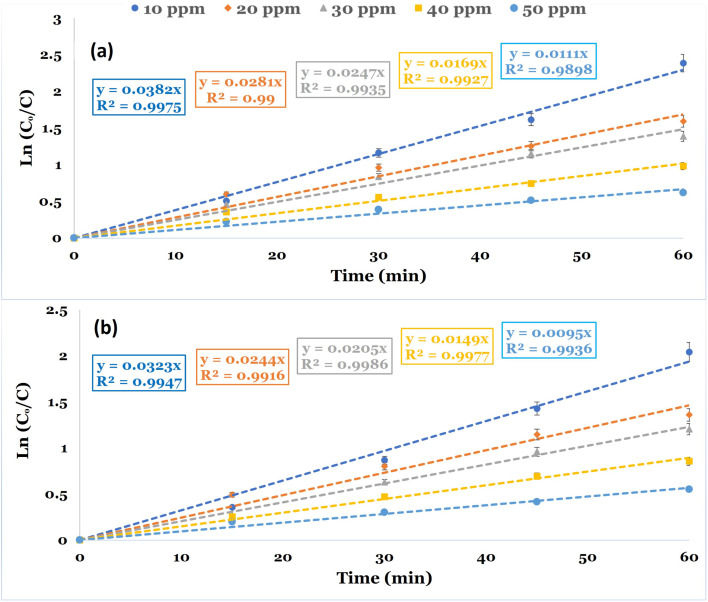
Linear plot of a pseudo-first-order kinetic model for the degradation of DCF at optimum reaction conditions (reaction condition: 0.1 L solution, [PDS]_o_ = 6 mM, catalyst doses = 0.75 g at neutral pH). (a) NCC and (b) CC.

Kinetic studies were conducted to evaluate the reaction rate constant values in the PDS-based AOPs for the removal of DCF. The studies were conducted utilizing CC and NCC catalysts at different DCF initial concentrations at neutral pH 6.8. The kinetic investigations are illustrated using the time dependency data. The pseudo-first-order rate equation was used to illustrate the degradation kinetics. Fig. 11a and b depicted the linear plot of ln(*C*_o_/*C*) *versus* the time of the degradation kinetics of DCF by NCC and CC catalysts. The results obtained showed that pseudo-first-order kinetic is the best model for describing the rate of DCF degradation by both catalysts with correlation coefficient values of *R*^2^ = 0.98–0.99. Table 2 also contains the constants of the pseudo-first order model, *K* (min^−1^), and the correlation coefficient *R*^2^. The experimental data obtained for both catalysts used in the Fenton-like degradation tests confirm that the pseudo-first-order equation is a suitable model for describing DCF degradation kinetics using NCC and CC catalysts. The determined rate constant and linearity of the plotting ln(*C*_o_/*C*) *versus* time (*t*) estimated by *R*^2^ are shown in Table 2. The results included the pseudo-first-order rate constant values and the *R*^2^ values at varied DCF concentrations. It is evident from the results that the degradation process of DCF by both NCC and CC catalysts followed pseudo-first-order rate kinetics and an increase in DCF concentrations caused the reduction in the rate constant values. The decrease in rate constant values with an increase in initial DCF concentration could be explained by an increased DCF/reactive oxygen species ratio with an increase in initial DCF concentration since the same concentration of reactive active species was expected to be generated under the same experimental conditions.

**Table 2:** Pseudo-first-order rate constants of NCC and CC catalysts under different initial concentrations of DCF at optimum conditions

Catalysts	DCF (mg L^−1^)	*K*_obs._ (min^−1^)	*R* ^2^
NCC	10	0.0382	0.9975
	20	0.0281	0.9900
	30	0.0247	0.9935
	40	0.0169	0.9927
	50	0.0111	0.9898
CC	10	0.0323	0.9947
	20	0.0244	0.9916
	30	0.0205	0.9986
	40	0.0149	0.9977
	50	0.0095	0.9936

### 3.4. Mechanism of metal based PDS activation, quenching test, and reusability of the catalysts

Among various methods of organics treatment, sulfate-based advanced oxidation processes have proven to be effective methods that decompose organic pollutants into harmless molecules such as water and CO_2_*via* the generation of hydroxyl and sulfate radicals from the catalytic activation of persulfates.^55^ PDS and PMS, also known as persulfates, are oxidants utilized in *in situ* chemical oxidation for the destruction of organic contaminants. Their peroxide bonds have low bond-dissociation energy, making them easily broken by a variety of mechanisms to produce sulfate and hydroxyl radicals through energy and electron transfer reactions.^56,57^ In this study, the catalytic activation of PDS was performed *via* NCC and CC catalysts synthesized from PCBs for the degradation and mineralization of pharmaceuticals DCF and IBP in an aqueous solution. The main active sites on catalyst-derived carbon-metal composite for PDS activation, according to previous research, include sp^2^ hybridized Carbon, oxygen-containing functional groups, transition metal species, persistent free radicals, internally or externally introduced doped heteroatoms, and carbon defects.^58,59^ The transition metal sites present in the catalyst can activate PDS for the production of SO4˙^−^*via* (R(3)), due to the strong electron-donating capacity of metal ions (M^*n*+^). The oxidized transition metal (M^(*n*+1)+^) could then take electrons from sulfate radical (R(4)) to ensure catalytic reusability. Additionally, transition metal promotes the production of persistent free radicals as active sites for PDS activation.^60,61^ Persistent free radical production mainly happens *via* electron transfer from quinone and phenol moieties to transition metal ions.

R3 S_2_O_8_^−2^ + M^*n*+^ → SO^−^_4_˙ + M^(n+1)+^ + SO^−2^_4_

R4 M^(*n*+1)+^ + SO^−^_4_˙ → M^*n*+^ + SO_4_^−2^

Oxygen functional groups on the surface of the catalyst, such as (–OH, –COOH), can activate PDS to produce sulfate radicals *via*R(5)–R(6).^62^

R5 S_2_O_8_^−2^ + –OOH → SO^−^_4_˙ + –OO˙ + HSO^−^_4_

R6 S_2_O_8_^-2^ + –OH → SO^−^_4_˙ + −O˙ + HSO^−^_4_

In general, neutral solutions (pH = 5.8 to 8.0) show the optimum performance of sulfate radical formation from PDS, and neutral pH also favors the electrostatic interaction between the surface-bound sulfate radicals and organic contaminants in the solution. An increase in pH over 8.0 may encourage sulfate radicals to oxidize one electron from hydroxyl, which causes sulfate radicals to transfer into less selective ˙OH *via*R(7). PDS activation in alkaline solution results in PDS hydrolysis and the production of HO_2_^−^, which then lowers PDS dissociation to sulfate radicals. Furthermore, in a highly alkaline solution, the sulfate radical can be further transformed to ˙OH.^63^ The hydrogen ion can scavenge sulfate radicals (R(8)) in an acidic solution (pH = 3.0).^64^

R7 H_2_O + SO^−^_4_˙ → ˙OH + HSO^−^_4_

R8 H^+^ + SO^−^_4_˙ → + HSO^−^_4_

The AOPs often show low removal efficiencies of target pollutants due to the high reactivity of hydroxyl radicals toward the naturally occurring organic matter that is present in natural water. However, because of the selective reactivity of the SO_4_˙^−^, sulfate-based AOPs are less impacted by the presence of background natural organic matter.^65,66^ Therefore, sulfate-based AOP has drawn more attention since it can effectively remove organic pollutants from water even when there are natural organic background materials present. The main mechanism by which the sulfate radical differs from the ˙OH is that it mainly reacts with pollutants by electron transfer.^67^

**Fig. 12:**
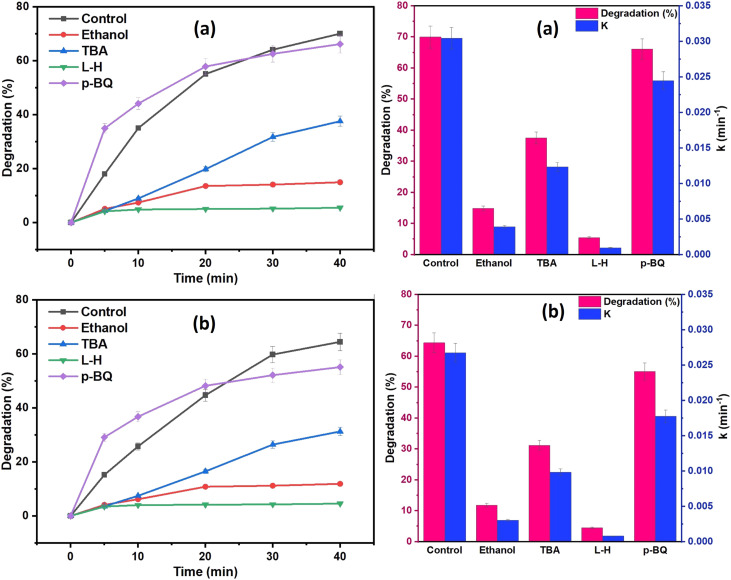
Effect of different scavenging agents (ethanol (EtOH), *tert*-butyl alcohol (TBA), *p*-benzoquinone (BQ), and l-histidine) on DCF degradation (a) NCC and (b) CC (reaction condition: 0.1 L solution, [DCF]_o_ = 20 mg L^−1^, [PDS]_o_ = 6 mM, treatment time = 40 min, catalyst doses = 0.75 g for CC or NCC).

To determine the dominant reactive oxygen species (ROS) involved in the degradation processes, a series of quenching tests were conducted on a 20 mg L^−1^ of DCF solution, and the results are shown in Fig. 12. Ethanol (EtOH) and *tert*-butyl alcohol (TBA) were the two most used scavengers for figuring out the contribution of the hydroxyl radical (˙OH) and sulfate radical (SO4˙^−^) in the oxidation process. TBA is more selective toward ˙OH than SO4˙^−^, whereas EtOH can react with both ˙OH and SO4˙^−.^^68^ EtOH, TBA, *p*-benzoquinone (BQ), and l-histidine (L-H) were used as the scavengers of both ˙OH and SO_4_˙^−^, ˙OH, O_2_˙^−^, and ^1^O_2_, respectively.^69^ From the results of Fig. 12, EtOH was shown to have a more pronounced inhibitory effect on DCF degradation when compared to TBA quenchers, showing that SO_4_˙^−^ and ˙OH were coexisting in the oxidation system and were responsible for DCF degradation, with SO_4_˙^−^ playing a more significant role than ˙OH. Additionally, as shown in Fig. 12, the DCF degradation efficacy was reduced with the addition of 20 mM BQ or L-H to the oxidation system from 70% to 5% and 100% using NCC, and from 60% to 4.5% and 52% using CC, respectively. These results demonstrated that ^1^O_2_ predominated the degradation process. Therefore, from the results of scavenging tests, it was determined that ^1^O_2_, SO_4_˙^−^, ˙OH, and O_2_˙^−^ all participated in the degradation processes. The results demonstrated that both radical and non-radical ROS contributed to the degradation processes since the generated ROS in the oxidation system by both catalysts was in the order of ^1^O_2_ > SO_4_˙^−^ > ˙OH.

From the results, it was predicted that in the presence of PDS/(NCC or CC) the radical and non-radical oxygen species in the order of (^1^O_2_ > SO_4_˙^−^ > ˙OH > O_2_˙^−^) were expected to be continuously generated in the oxidation system, which decomposes the pharmaceutical pollutants (DCF and IBP) into degradation by-products, which are then gradually mineralized into water, carbon dioxide and mineral acids.

**Fig. 13:**
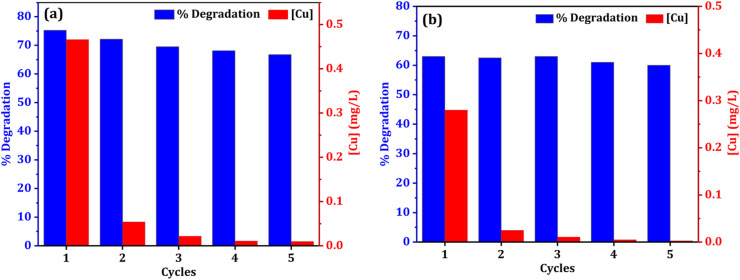
Reusability and leaching copper ion of both catalysts (a) NCC and (b) CC (reaction condition: 0.1 L solution, [DCF]_o_ = 20 mg L^−1^, [PDS]_o_ = 6 mM, treatment time for each cycle = 1 hour, catalyst doses = 0.75 g for CC or NCC).

The stability and reusability of catalyst is always a concern in heterogeneous catalysis system. In terms of practicality and cost, catalyst reusability is critical. To assess a catalyst's performance, the stability of recycling is an important consideration. The reusability of both catalysts (NCC and CC) was tested for five cycles to examine the catalyst stability in the degradation of DCF (Fig. 13). After the treatment reactions, the used CC or NCC catalysts were collected and washed several times with deionized water and dried overnight, and then used for another cycle. Fig. 11 shows that the DCF removal efficiency only decreased by 8.5% and 3% after five subsequent cycles using NCC and CC, respectively. As shown in Fig. 13, both catalysts achieved effective DCF degradation after five cycles with no significant decreases in catalytic activity when compared to the first run, indicating that both catalysts are reusable and have excellent stability for practical applications. Previous research suggested that DCF degradation could be caused by catalytic interactions between metals in PCB catalysts and PDS, which produce sulfate radicals as an oxidant in the degradation processes. NCC and CC catalysts contain copper, iron, aluminum, zinc, and nickel. Because the main metal in both catalysts is copper, the leached copper in the reaction system is critical to evaluating the contamination of treated solutions with heavy metals and the contribution of homogeneous catalytic activation of PDS. ICP-OES was used to determine the amount of metal leaching in the treated solution after each cycle and the results are shown in Fig. 11a and b. It was interesting to note that the concentration of metal ion leaching after treatment processes was very low and within the acceptable range of WHO permitted drinking water parameters.^70^ In the first cycle, the highest concentration of copper ion leaching was measured to be 0.466 and 0.28 mg L^−1^ after 1 h, using NCC and CC, respectively (Fig. 13). It was observed that the concentration of copper ions was significantly reduced with practically the same rate of degradation after the first cycle, indicating that the heterogeneous catalytic activation of PDS was caused by both catalysts. The concentration of other metal ions, such as Fe, Co, Ag, Zn, Al, and Ni, was also measured after each cycle, and the detected values were lower than the detection limit, indicating that these metal ions had not been leached.

## 4. Conclusions

The key to sustainable development is proper waste disposal. Electronic waste can be valued to fabricate catalysts that can be used in sulfate radical based AOPs to activate persulfate. In this work two PDS activator catalysts (NCC and CC) have been prepared successfully *via* thermal treatment under air and nitrogen pyrolysis, using scrap PCBs as raw materials. The fabricated catalyst showed excellent catalytic activity toward the removal of pharmaceutical DCF and IBP from water in the presence of PDS at circumneutral pH. The synthesized catalysts could be recycled and reused after application with excellent stability, indicating that the prepared catalyst has promising potential as a PDS activator catalyst in practical application. Scavenging studies showed that ^1^O_2_ > SO_4_˙^−^ > ˙OH played major roles in the degradation processes. This research provides important and promising waste-to-resource strategy instructions for the easy design and manufacturing of a highly efficient and reusable PDS activator catalyst from scrap PCBs for application in water remediation from pharmaceutical contaminants. Conceptually, large-scale installations based on the suggested approach would be both environmentally and economically sustainable. The concentration of leaching metal ions in the treated solution is within the acceptable range of WHO permitted drinking water parameters, which is an interesting result for practical applications. The proposed NCC/PDS and CC/PDS systems offered significant promise for practical application in the removal of pharmaceutical contaminants. However, more research is needed to validate its applicability in engineering practice.

## Conflicts of interest

There are no conflicts to declare.

## Supplementary Material

RA-013-D2RA07263G-s001
