# Supplementary material for: Heterogeneous catalytic activation of peroxydisulfate toward degradation of pharmaceuticals diclofenac and ibuprofen using scrap printed circuit board
Source: RSC Adv. 2022 Dec 20;13(1):115–28. doi: 10.1039/d2ra07263g (PMC9764427; doi:10.1039/d2ra07263g)
Supplement: RA-013-D2RA07263G-s001 [file RA-013-D2RA07263G-s001.pdf]

Electronic Supplementary Material (ESI) for RSC Advances.
This journal is © The Royal Society of Chemistry 2022

Supplementary Information

Heterogeneous catalytic activation of peroxydisulfate toward degradation of pharmaceutical diclofenac and ibuprofen using scrap printed circuit board

Kosar Hikmat Hama Aziz[1,2,3]

[1]*Department of Chemistry, College of Science, University of Sulaimani, Qlyasan Street Sulaimani City 46001, Kurdistan Region, Iraq*

[2]*Department of Medical Laboratory of Science, College of Health Sciences, University of Human Development, Sulaimani, Iraq*

* *Corresponding author's email: kosar.hamaaziz@univsul.edu.iq*

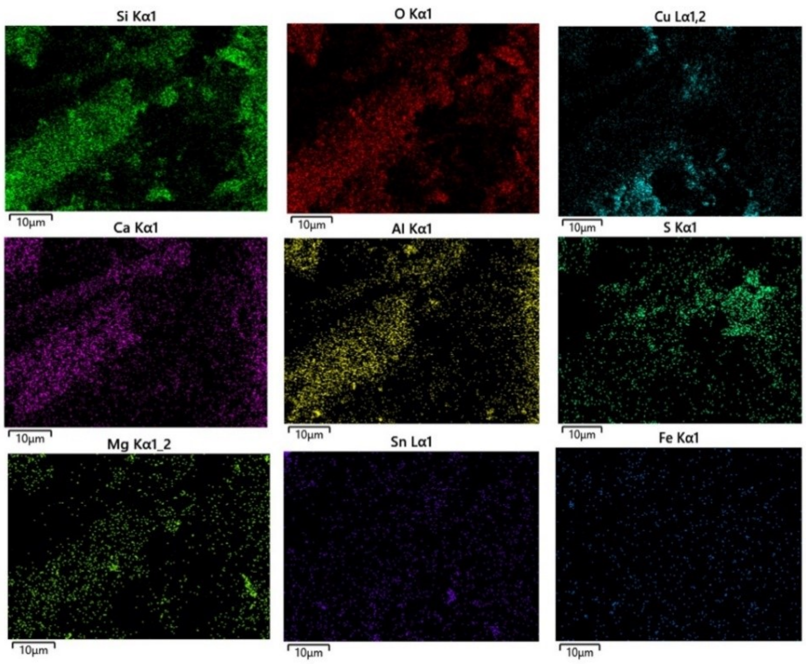

Fig. S1. SEM-EDS elemental mapping of NCC

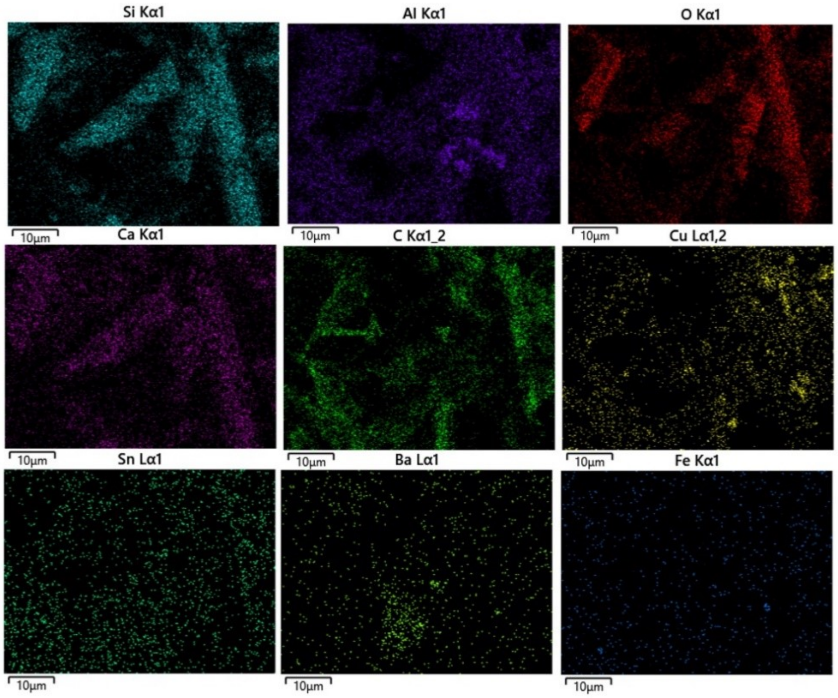

Fig. S2. SEM-EDS elemental mapping of CC

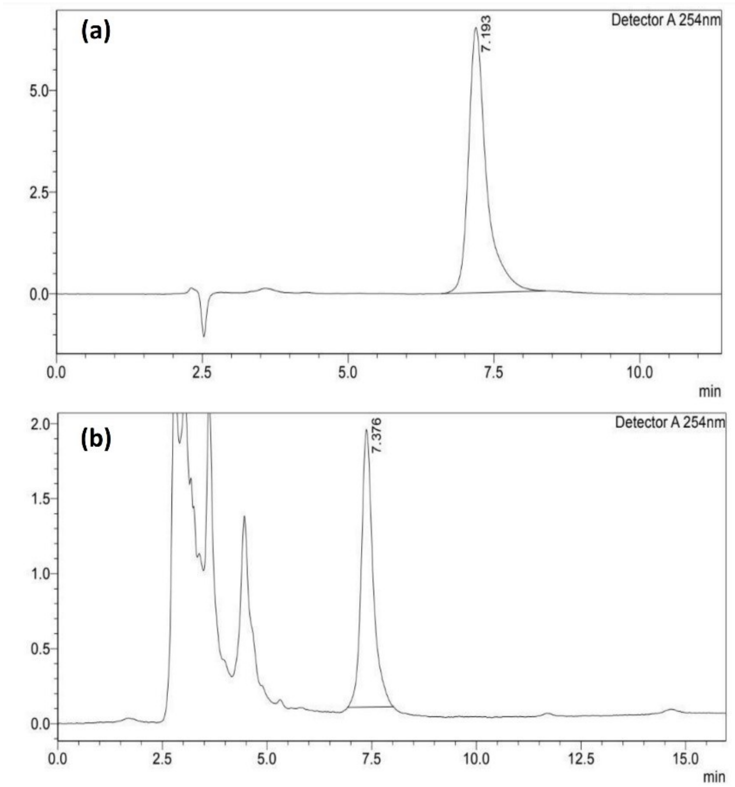

Fig. S3. Chromatogram of DCF, (a) before degradation (b) after degradation

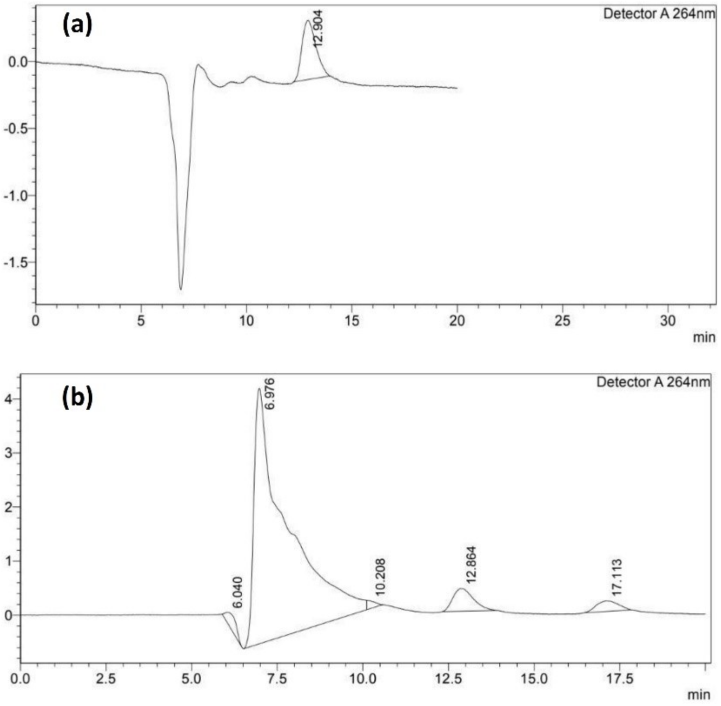

Fig. S4. Chromatogram of IBP, (a) before degradation (b) after degradation

Table S1. The chemical composition of both catalysts is detailed in the XPS results.

C-PCB

Element's symbol	Peak binding energy (ev)	Element's state
Br(3d)	69.08	CH_3Br, organic Br, metal Br
Si(2p)	103.2	SiO_2, metal SiO_4
C(1s)	285	C-C or C-H, carbonate
Ca(2p)	348.55	$CaCO_3$, CaO element
Sn(3d)	487.06	SnO, SnO_2 element
O(1s)	532.31	SiO_2, metal CO_3, metal oxide
Fe(2p)	716.02	Element
Co(2p)	781	CoO, Co_2O_3, Co_3O_4 element
Cu(2p)	934.97	CuO element
Mg(1s)	1305.05	MgO element

Metal oxide (CaO, Co_2O_3, Co_3O_4, CoO, SnO, CuO, Cu_2O, MgO)

Elemental (Fe, Sn, Co, Mg, Cu)

NC-PCB

Element's symbol	Peak binding energy (ev)	Element's state
Si(2p)	103.4	SiO_2, metal SiO_4
S(2P)	169.74	$CaSO_4$, metal SO_4, metal SO_3, organic SO_2
C(1S)	285.14	C-C or C-H, carbonate
Ca(2p)	348.08	$CaCO_3$, CaO element
Sn(3d)	486.89	SnO, SnO_2 element
O(1S)	531.94	SiO_2, metal CO_3, metal oxide
Fe(2p)	715.35	Fe_2O_3 element
Co(2p)	780.89	CoO, Co_2O_3, Co_3O_4 element
Cu(2p)	934.4	CuO element
Mg(1s)	1304.72	MgO element

Metal oxide (CaO, Co_2O_3, Co_3O_4, CoO, SnO, CuO, Cu_2O, MgO, Fe_2O_3)

Elemental (Fe, Sn, Co, Mg, Cu)

Table S2: Leaching metal ions from catalysts in the treated solution

Time(min)	Fe(ppm)	Ba(ppm)	Ag(ppm)	Al(ppm)	Cr(ppm)	N(ppm)	Zn(ppm)
0	0	0	0	0	0	0	0
5	L.D	0.479	L.D	L.D	L.D	L.D	L.D
15	L.D	0.627	L.D	L.D	L.D	L.D	L.D
25	0.0215	0.712	L.D	L.D	L.D	L.D	L.D
40	0.142	0.825	L.D	L.D	L.D	L.D	L.D
55	0.266	0.952	L.D	L.D	L.D	L.D	L.D
70	0.492	1.02	L.D	L.D	L.D	L.D	L.D

Acknowledgments

The author thanks the University of Sulaimani, Kurdistan-Iraq, for supporting the work and acknowledging the help of Razga Company in Kurdistan-Iraq for this research.

Conflicts of Interest

The authors declare no conflict of interest.
